# Building, testing, and learning from network models of human aging

**DOI:** 10.1093/geroni/igaa057.1576

**Published:** 2020-12-16

**Authors:** Andrew Rutenberg, Spencer Farrell, Arnold Mitnitski, Kenneth Rockwood, Garrett Stubbings

**Affiliations:** Dalhousie University, Halifax, Nova Scotia, Canada

## Abstract

We have developed computational models of human aging that are based on complex networks of interactions between health attributes of individuals. Our “generic network model” (GNM) captures the population level exponential increase of mortality with age in Gompertz’s law together with the exponential decrease of health as measured by the frailty index (FI). Our GNM includes only random accumulation of damage, with no programmed aging. Our GNM allows large populations of model individuals to be quickly generated with detailed individual health trajectories. This allows us to explore individual damage propagation in detail. To facilitate comparison with observational data, we have also developed and tested new approaches to binarizing continuous-valued health data. To extract the most information out of available cross-sectional or longitudinal data, we have also reconstructed interactions from generalized network models that can predict individual health trajectories and mortality.

